# Climate Change and Sustainable Healthcare: Knowledge, Attitudes, and Educational Role of Healthcare Workers

**DOI:** 10.3390/healthcare14111576

**Published:** 2026-06-04

**Authors:** Vincenza Sansone, Giovanna Paduano, Fabrizio Liguori, Francesca Gallè, Concetta Paola Pelullo

**Affiliations:** 1 Department of Experimental Medicine, University of Campania “Luigi Vanvitelli”, Via Luciano Armanni 5, 80138 Naples, Italy; vincenza.sansone@unicampania.it (V.S.); giovanna.paduano@unicampania.it (G.P.); 2 Department of Medical, Human Movement and Well-Being Sciences, University of Naples “Parthenope”, Via Medina 40, 80133 Naples, Italy; fabrizio.liguori@collaboratore.uniparthenope.it (F.L.); francesca.galle@uniparthenope.it (F.G.)

**Keywords:** attitude, climate change, cross-sectional study, education, health, healthcare workers, knowledge, nurses, physicians, practice, sustainable healthcare, sustainability

## Abstract

**Background:** The role of healthcare workers (HCWs) is crucial in promoting and educating about sustainable behaviors. This study aimed to assess Italian HCWs’ knowledge, attitudes, practices, and educational role regarding climate change and its health implications. **Methods:** A cross-sectional study was conducted from May to December 2024. **Results:** Among the 564 HCWs who participated, 45% and 40.3% considered climate change very important and urgent, respectively. Nurses, who had at least one chronic disease, who self-assessed their knowledge of climate change as good/very good, who needed additional information, and those who knew that problems in global food supply are consequences of climate change were more likely to consider it an urgent problem. Women, those married/cohabitant, and who knew that the spread of infectious diseases, problems in the global food supply, water scarcity or clean water conservation were consequences of climate change, were more likely to believe that climate change is causing health problems. Men, who had at least one chronic disease, who knew that infants/children, elderly and people with multiple medical conditions are more sensitive to climate change, those very scared of climate change, and who received information in training courses were more likely to educate patients for improving sustainability and health protection. **Conclusions:** Tailored training and integrating sustainability for HCWs could significantly support the health sector in adapting in climate change mitigation.

## 1. Introduction

Climate change represents a major threat to human health, healthcare systems, and environmental sustainability worldwide. Rising of average temperatures, the increasing frequency and intensity of extreme weather events such as floods, wildfires, and heatwaves, as well as the spread of communicable diseases (e.g., vector-borne diseases like dengue and malaria), the worsening of respiratory and cardiovascular diseases, and the increase in stress-related mental health problems are just some of the direct and indirect effects of this phenomenon [1]. According to the World Health Organization (WHO) [2], climate change is expected to cause approximately 250.000 additional deaths between 2030 and 2050, due to these causes. Moreover, these changes can negatively affect healthcare resources and infrastructures, exacerbating existing inequalities and challenges in healthcare systems [3], highlighting the need for an urgent Public Health response.

In this complex scenario, the role of healthcare workers (HCWs) is crucial not only in managing climate emergencies and related diseases, but also in promoting sustainable behaviors and raising community awareness of environmental risks. Indeed, HCWs are key figures capable of positively influencing individual and collective choices, contributing to the dissemination of preventive practices and more sustainable lifestyles [4,5]. The role of HCWs in addressing climate change is increasingly recognized within global Public Health frameworks. Indeed, the importance of healthcare professionals is recognized not only in clinical care but also in promoting health education, prevention strategies, and sustainable healthcare systems [6,7,8]. In this context, climate change is considered not only as an environmental issue, but as a key determinant of health, requiring the integration of climate-related competencies into healthcare education and professional practice.

Knowledge, attitudes, and behaviors of HCWs regarding environmental issues, as well as the adaptation strategies available in healthcare facilities, often depend on their level of training and resource availability [9].

In the literature, research has been conducted on HCWs’ knowledge, attitudes, and practices about environmental issues [10,11]. However, despite their important role, healthcare professionals’ knowledge and attitudes regarding the health implications of climate change remain insufficiently developed. Addressing climate change-related knowledge gaps among HCWs is essential to enhance their professional skills and practices, strengthen the role of the healthcare system in combating climate change, and foster an integrated and sustainable approach to healthcare that effectively addresses environmental challenges [12,13]. Although several studies have explored HCWs’ knowledge and attitudes towards climate change in different countries [9,14,15], evidence from Italy remains limited and fragmented. Indeed, existing Italian studies [16,17,18] have primarily focused on specific professional groups or dimensions, such as awareness or attitudes, without providing a comprehensive assessment that includes knowledge, attitudes, and sustainable practices. Therefore, these important aspects, such as the role of education and perceived preparedness, remain underexplored. Moreover, few studies have analyzed the educational role of HCWs, especially nurses, in promoting sustainability and enhancing patient awareness, which has received limited attention in the literature [19,20].

Given the susceptibility of Italy to climate change [21], this study aimed to comprehensively assess Italian HCWs’ knowledge, attitudes, and practices regarding climate change and its health implications, as well as their perception about educational needs and role in promoting sustainable healthcare behaviors.

## 2. Materials and Methods

### 2.1. Study Design and Setting

This cross-sectional study was conducted from May to December 2024, in Naples, Italy. HCWs were recruited through a convenience sampling approach using email and social media. The exact number of HCWs who received the invitation could not be determined, as the survey link was disseminated through multiple digital channels.

### 2.2. Data Collection

HCWs were invited to participate through email and social media. The invitation contained a link to a private Telegram channel. Each participant, once they accessed the Telegram channel, read an information sheet describing the objectives of the study. Participants provided informed consent electronically before accessing the questionnaire by confirming their willingness to participate after reading the study information sheet. They were only allowed to respond once. No incentives were offered for completing the survey. Data confidentiality was ensured by collecting responses anonymously, without recording any personally identifiable information. Data were stored securely, protected with a password, and accessed only by the research team.

### 2.3. Questionnaire

A review of the literature was conducted to identify existing questionnaires and key domains related to climate change and health, including knowledge, attitudes, and practices among HCWs. Although no fully standardized instrument exists for all domains assessed, an ad hoc questionnaire was developed to collect data on knowledge, attitudes, and behaviors on climate change among HCWs [14,15,19,22,23]. The self-administered questionnaire included variables such as socio-demographic characteristics, knowledge of climate change causes and consequences, attitudes towards climate change, self-reported practices, and sources of information.

The first part comprised questions collecting socio-demographic, anamnestic, and professional data, including age, gender, marital status, education level, presence of at least one chronic disease, professional role, and ward type.

The second section consisted of questions evaluating HCWs’ knowledge about climate change, causes, and consequences. Responses were recorded as “yes,” “no,” or “do not know”. The third section assessed attitudes towards climate change through statements related to the perception of this phenomenon as an inevitable consequence of our society, its negative effects on the Italian population, human activities as the main cause, etc. Moreover, in this section, attitudes on climate change and its consequences on the future health needs of the population and its role in various health-related problems (such as infectious diseases, mental health, respiratory disease, etc.) were also investigated. These items were rated on a 5-point Likert scale, from “strongly disagree” to “strongly agree.” Finally, it was assessed how important the HCWs considered several interventions to reduce climate change, such as controlling greenhouse gas emissions, creating public health emergency plans, formulating and implementing laws and regulations, and improving scientific research. These items were rated on a 5-point Likert scale, from “not at all important” to “very important”.

The fourth part investigated the HCWs’ practices regarding climate change, asking which behaviors they believed were useful and, among these, which they had effectively implemented. Responses were recorded as “yes,” “no,” or “do not know”. Moreover, participants were asked to report the frequency with which they adopt eco-sustainable actions, such as using public transport, walking or cycling to work, etc., during their working activity on a 5-point Likert scale, from “always” to “never”.

The fifth part explored sources of information and perceived informational needs related to climate change for HCWs.

A pilot study was conducted with 100 HCWs to assess the feasibility of the survey, the clarity and readability of questions, and the reliability of the instrument. The pilot study indicated acceptable internal consistency and clarity of the questionnaire items. The internal consistency of the questionnaire was evaluated by calculating Cronbach’s alpha coefficient. The pilot study confirmed the instrument’s reliability, yielding a scale reliability coefficient of 0.903, indicating excellent internal consistency for assessing HCWs’ knowledge, attitudes, and behaviors.

The study protocol and the questionnaire received approval from the Ethics Committee of the University of Campania “Luigi Vanvitelli” (approval number 0018199/i/01.07.2024).

### 2.4. Data Analysis

Data analysis was performed using Stata version 17 (StataCorp, College Station, TX, USA) [24]. Descriptive statistics, including frequencies, means, and standard deviations, were calculated to summarize the characteristics of the study population. The models were developed according to Hosmer and Lemeshow’s strategy [25] that includes the following steps: (1) bivariate analysis of each variable considered, using the appropriate test statistic (chi-square test, and Student’s *t*-test); (2) choice of the way to include independent variables in the model (continuous or dichotomous) by taking into account how each of these ways better fitted the data at the bivariate analysis; (3) inclusion in the model of any variable whose bivariate test had a value of *p* ≤ 0.25 or that was judged to potentially have an influence on the investigated outcomes; (4) the values for variables’ entry and removal in the final models were, respectively, *p* = 0.2 and *p* = 0.4 throughout the stepwise selection procedure. Model fit was assessed using log-likelihood statistic. The multivariate regression models were performed to identify factors associated with the following outcomes of interest: self-assessed knowledge about climate change (dichotomous) (poor/low/moderate = 0; good/very good = 1) (Model 1); considering climate change an urgent problem (dichotomous) (no = 0; yes = 1) (Model 2); believing that climate change will negatively affect global health (dichotomous) (strongly disagree/disagree/uncertain/agreed = 0; strongly agree = 1) (Model 3); believing that climate change is causing health problems (dichotomous) (no = 0; yes = 1) (Model 4); believing information campaigns and Public Health emergency plans very important to reduce climate change (dichotomous) (no = 0; yes = 1) (Model 5); educating patients for improving sustainability and health protection (dichotomous) (no = 0; yes = 1) (Model 6). The independent variables included in the final models are shown Supplementary File S1.

The results are presented as odds ratios (ORs) and 95% confidence intervals (CIs). A value of *p* ≤ 0.05 was judged to be statistically significant, and all reported values are two-tailed.

## 3. Results

Out of a total of 738 HCWs who accessed the Telegram channel, 564 HCWs agreed to participate, resulting in an overall response rate of 76.4%. The socio-demographic, anamnestic, and professional characteristics of the participants are summarized in Table 1. The average age was 39.9 years (range 23–65), with 56.6% identifying themselves as female; 51.7% were married, and 19% had at least one chronic disease. Regarding work settings, the majority (73.1%) were nurses, and 22.3% of participants worked in an emergency/resuscitation/intensive care ward (Table 1).

Self-assessed knowledge about climate change was defined to be moderate by 40% and good by 26.2% of participants.

The results of the multivariate regression model showed that men, older HCWs, and those who did not need additional information on climate change were more likely to self-assess their knowledge of climate change as good/very good (Model 1 in Table 2).

Moreover, 45% and 40.3% considered climate change to be very important and urgent, respectively. The results of the multivariate regression model showed that nurses, those who had at least one chronic disease, those who self-assessed their knowledge of climate change as good/very good, those who needed additional information on climate change, and those who knew that problems in the global food supply are consequences of climate change were more likely to consider climate change an urgent problem (Model 2 in Table 2).

When asked about climate change causes, HCWs indicated serious air pollution (85.5%), destruction of forest and farmland (68.3%), and the greenhouse effect (58.7%) as the main causes (Table 3).

Most of participants identified rising temperatures (84.9%), melting glaciers and rising sea levels (81.9%), and extreme weather events (67.5%) as the main consequences of climate change (Table 3).

More than half of participants indicated infants/children (64.4%), elderly (62.9%), people with low social status (41.5%), people with multiple medical conditions (38.1%), and adolescents (37.9%), as the categories who are most sensitive to climate change.

When HCWs were asked about their attitudes towards climate change, the respondents strongly agree/agree that climate change is happening (85.5%), that climate change will have negative effects on the Italian population (82.2%), and that it is inevitable for our society (55.8%).

Furthermore, the HCWs were asked to indicate their level of agreement/disagreement with some statements on climate change, its consequences on the future health needs of the population, as a cause of future health-related problems, and on actions to reduce it (Supplementary File S2). In particular, 78.9%, 59%, and 58,9% strongly agree/agree that climate change will cause global health problems, that it will be a problem of care relevance for HCWs, and the issue should be included in HCWs’ training, respectively.

The results of the multivariate regression model showed that HCWs who self-assessed their knowledge of climate change as good/very good, those who knew that the spread of infectious diseases is a consequence of climate change, and those who knew that problems in the global food supply are consequences of climate change were more likely to strongly agree that climate change will negatively affect global health (Model 3 in Table 2).

Regarding the health-related impacts of climate change, most respondents strongly agree/agree that air-quality-related illnesses (83%), diarrhea (71.5%) caused by foodborne or waterborne diseases following floods or inundations (salmonella, giardia, etc.), mental health conditions (70.9%), and vector-borne diseases (68.4%), increased because of climate change (Supplementary File S2). The results of the multivariate regression model showed that women, those who were married/cohabitant, and those who knew that the spread of infectious diseases, the problems in the global food supply and the water scarcity or clean water conservation were consequences of climate change were more likely to believe that climate change is causing health problems (Model 4 in Table 2).

Moreover, the HCWs were asked how important they considered some interventions to reduce climate change. Information campaigns (86.8%), formulating and implementing laws and regulations related to the fight against climate change (85.8%), and improving scientific research on useful interventions to address climate change (84.4%) were identified as important/very important interventions to reduce climate change (Supplementary File S2).

The results of the multivariate regression model showed that women, elderly HCWs, those who knew that problems in the global food supply were consequences of climate change, and those who knew that infants/children, elderly and people with multiple medical conditions are more sensitive to climate change were more likely to believe that information campaigns and Public Health emergency plans are very important to reduce climate change (Model 5 in Table 2).

**Figure 1:**
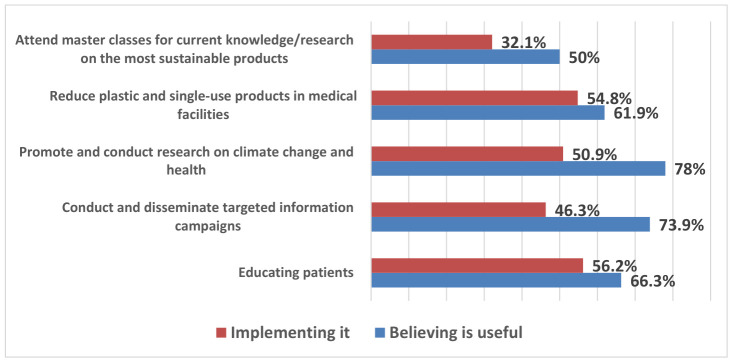
Actions for improving sustainability and health protection.

Promote and conduct research on climate change and health (78%), conduct and disseminate targeted information campaigns (73.9%), and education of patients (66.3%) were identified as useful actions to improve sustainability, but the implementation of these actions was less reported by participants (Figure 1).

The results of the multivariate regression model showed that men, those who had at least one chronic, those who knew that infants/children, elderly and people with multiple medical conditions are more sensitive to climate change, those who were very scared of climate change, and those who received information about climate change in training courses were more likely to educate patients for improving sustainability and health protection, whereas those with a master’s degree/PhD were less likely to do so (Model 6 in Table 2).

Table 4 reports the frequency with which HCWs adopt eco-sustainable behaviors during their daily life.

Finally, 70% of HCWs reported having received information about climate change from the Internet, followed by television (65.8%), and journals (38.8%). Additionally, 79.8% expressed an interest in obtaining more information on climate change.

## 4. Discussion

This study contributes to the literature about knowledge, attitudes, and practices of HCWs regarding climate change and its health implications, providing important insights into the field, especially considering the vulnerability of Italy and the Italian population to climate change. It highlights a gap between awareness and the actual implementation of sustainable behaviors among HCWs. These findings should be interpreted within the broader recognition of climate change as a key determinant of population health and an emerging domain of healthcare professional competence.

The high proportion of HCWs who acknowledge climate change as an urgent problem testifies to their increased awareness of this issue and aligns with existing literature [22]. This evidence underscores an emerging consensus on the need for proactive engagement about climate.

Our multivariate regression analyses reveal several significant predictors that influence HCWs’ self-assessment of their level of knowledge. Gender differences emerged in perceptions and self-assessment. Men were less likely to consider their knowledge as good or very good, in contrast with existing literature, which typically indicates that women often demonstrate greater environmental concern and awareness [26,27].

The moderate self-assessed level of knowledge about climate change observed in our sample agrees with findings from other similar studies, which identified variability in climate health literacy among healthcare professionals [22]. The association between older age and self-perceived higher knowledge levels with greater awareness of climate change urgency and importance may be due to experience, exposure to environmental topics over time, and vulnerability of older individuals, which also increases the risk of adverse health effects due to climate change [28]. The self-perceived moderate level of knowledge reported in the results indicates opportunities for enhancement, as in other studies where HCWs often had only a basic understanding of the climate–health relationship [29,30]. This underscores the need for targeted educational programs to enhance HCWs’ climate literacy, especially among younger or less-informed HCWs, in order to improve their capacity to respond effectively; indeed, better training could enable health workers to better fulfill their role as educators on environmental health issues [16,31]. From a practical perspective, improving HCWs’ knowledge through structured training programs could enhance their ability to recognize climate-related health risks and respond appropriately in clinical settings.

Knowledge of climate change consequences significantly predicted perceptions of its health impacts, recognizing problems in the global food supply and understanding that vulnerable groups increased the likelihood of perceiving climate change as a health threat. By highlighting the importance of health-related climate effects in building perceptions, these findings are consistent with the literature [6,32]. Furthermore, the gap between HCWs’ positive attitudes on climate change mitigation and their current practices suggests that awareness alone is insufficient to drive behavioral change, and that institutional support, clear guidelines, and organizational strategies are necessary to facilitate the adoption of sustainable practices in healthcare settings. Although a majority consider actions like promoting and conducting research on climate change and health, and disseminating targeted information campaigns as useful, their engagement in these actions remains relatively low. This gap is consistent with findings from other studies and suggests that certain barriers, such as the lack of institutional support, can make it difficult the transition of knowledge into practice despite awareness and positive attitudes [22]. Therefore, healthcare institutions should integrate sustainability into their policies, providing resources and incentives for HCWs to adopt sustainable behaviors [33].

The results suggest that higher education may promote critical awareness, but also highlight potential gaps in climate change education within advanced curricula. Additionally, concern about climate change influenced professional preparedness. This aligns with studies that demonstrated how concern and formal education are critical motivators for HCWs to engage on climate issues [34]. These findings highlight the importance of integrating climate change education into both undergraduate and continuing professional development programs, with a focus on practical competencies rather than purely theoretical knowledge. These findings underscore the necessity of integrating climate change and sustainability into healthcare education. Moreover, education should transition from theoretical approaches to competency-based frameworks that prioritize sustainable clinical practice, more efficient resource management, and patient empowerment, thus fostering institutional change beyond individual behavioral modification.

The Internet and television are the main sources of information used by HCWs, highlighting the importance of accessible and tailored educational resources. Given they expressed high interest in receiving further information, there exists an opportunity for health authorities to develop targeted training information, awareness campaigns, and education programs on climate and health [35]. Such initiatives could lead to more informed and proactive HCWs capable of better managing climate-related health challenges. Developing standardized and evidence-based educational resources could improve the quality and consistency of information accessed by HCWs.

Our study also emphasizes the role of HCWs as active participants in climate change mitigation and adaptation strategies. HCWs, with the necessary knowledge and resources to lead climate-health initiatives, could influence patient behaviors, advocate for policy changes, and contribute to community resilience [4]. Indeed, from a policy perspective, establishing national climate-health competency frameworks is essential to standardize HCWs training and reduce disparities in preparedness, while interprofessional education can enhance collaborative sustainability efforts and strengthen multidisciplinary teams’ role in climate adaptation and mitigation [36]. In this context, HCWs can act as key agents of change, not only in clinical practice but also in community education and policy advocacy. Strengthening this role requires institutional recognition and support.

Despite most HCWs recognized the importance of different actions to mitigate the impact of climate change, their current implementation of eco-sustainable behaviors remains limited. Barriers such as a lack of resources and institutional policies, and cultural factors may delay the adoption of sustainable practices, underlining the need for institutional support. From a policy and practice perspective, these findings suggest the need for concrete actions, including the integration of sustainability into healthcare curricula, the development of targeted training programs, and the implementation of institutional policies that promote environmentally sustainable practices.

Several limitations should be acknowledged when considering these results. Given the design of the cross-sectional study, it is difficult to determine cause-effect relationships among the examined variables and the different outcomes of interest. Additionally, self-reported data may introduce social desirability bias, which potentially overestimated attitudes and current practices. Moreover, the use of a Telegram channel for recruitment may have introduced selection bias, as participation required access to and familiarity with digital platforms, potentially excluding less digitally engaged HCWs. Finally, the use of a convenience sampling method for data collection via email and social media introduces a potential self-selection bias, as HCWs with a pre-existing interest in climate change or those who are more digitally active were more likely to participate. Consequently, the findings may not be fully representative of the entire HCWs, and caution should be exercised when generalizing these results to broader contexts, also considering that questionnaires were collected in a single-city setting.

## 5. Conclusions

In conclusion, while HCWs demonstrate awareness of climate change and its health impacts, gaps in knowledge and implementation of sustainable practices remain. Strengthening climate-related education and integrating sustainability into healthcare training and policies are essential to translate awareness into action. Concrete actions should include integrating sustainability into HCWs curricula, developing targeted training programs, and promoting institutional policies that encourage sustainable practices within healthcare settings.

## Figures and Tables

**Table 1:** Participants’ socio-demographic, anamnestic and professional characteristics.

Characteristics	N	%
*Gender*		
Female	319	56.6
Male	227	40.2
Non-binary	18	3.2
*Age*, *years*	39.9 ± 11.3 (23–65) *	
*Marital status*		
Married/Cohabitant	291	51.7
Unmarried/separated/divorced/widowed	272	48.3
*Education*		
Degree	346	61.3
Master’s degree/PhD	218	38.6
*Professional role*		
Nurses	412	73.1
Physicians	57	10.1
Nursing supporting staff	55	9.7
Others (physiotherapist, biologist, etc.)	40	7.1
*Ward type*		
Clinical Departments (pediatric, cardiology, etc.)	386	68.5
Emergency/Resuscitation/Intensive care	126	22.3
Surgery/Operating Room	52	9.2
*Chronic disease*		
No	457	81
At least one	107	19

* Mean ± Standard Deviation (Range).

**Table 2:** Multivariate logistic regression models to identify those factors predicting the outcomes of interest.

Variable	OR	95% CI	*p*
**Model 1. Self-assessed knowledge about climate change** Log likelihood = −301.32598, χ^2^ = 35.48 (4 df), *p* < 0.0001 (sample size 544)			
Elderly HCWs	1.03	1.01–1.05	0.001
Males	0.59	0.40–0.88	0.010
Not needing additional information on climate change	0.55	0.35–0.86	0.010
Receiving information about climate change from scientific journals	1.33	0.89–1.97	0.154
**Variable**	**OR**	**95% CI**	***p***
**° Model 2. Considering climate change an urgent problem** Log likelihood = −318.2977, χ^2^ = 98.82 (10 df), *p* < 0.0001 (sample size 546)			
Good/very good self-assessed knowledge about climate change	3.83	2.48–5.91	<0.001
Having at least one chronic disease	2.31	1.43–3.73	0.001
Knowing that problems in the global food supply were consequences of climate change	1.84	1.22–2.76	0.003
Needing additional information on climate change	2.03	1.21–3.44	0.008
Nurses	1.58	1.01–2.48	0.046
*Ward type*			
Emergency/Resuscitation/Intensive care	Backward elimination		
Surgery/Operating Room	0.54	0.27–1.07	0.078
Clinical Departments	Reference category		
Knowing that the spread of infectious diseases was a consequence of climate change	1.45	0.95–2.21	0.082
Receiving information about climate change from scientific journals	1.38	0.96–2.04	0.108
Females	1.31	0.87–1.96	0.191
Knowing that infants/children, the elderly, and people with multiple medical conditions are more sensitive to climate change	1.22	0.79–1.88	0.378
**Variable**	**OR**	**95% CI**	***p***
**Model 3. Believing that climate change will negatively affect global health** Log likelihood = −346.05025, χ^2^ =48.55 (9 df), *p* < 0.0001 (sample size 546)			
Good/very good self-assessed knowledge about climate change	1.68	1.12–2.51	0.012
Knowing that the spread of infectious diseases was a consequence of climate change	1.61	1.08–2.41	0.019
Knowing that problems in the global food supply were consequences of climate change	1.54	1.04–2.27	0.029
Females	1.42	0.97–2.07	0.068
Knowing that water scarcity or clean water conservation were consequences of climate change	1.57	0.96–2.56	0.073
Having a postgraduate degree (master, PhD)	1.39	0.96–2.01	0.077
Knowing that infants/children, elderly, and people with multiple medical conditions are more sensitive to climate change	1.31	0.87–1.99	0.198
Needing additional information on climate change	1.34	0.83–2.16	0.228
Having at least one chronic disease	1.31	0.83–2.07	0.242
**Variable**	**OR**	**95% CI**	***p***
* Model 4. Believing that climate change is causing health problems Log likelihood = −320.14178, χ^2^ = 45.39 (8 df), *p* < 0.0001 (sample size 545)			
Knowing that water scarcity or clean water conservation were consequences of climate change	1.95	1.19–3.19	0.008
Females	1.68	1.13–2.51	0.011
Knowing that the spread of infectious diseases was a consequence of climate change	1.69	1.11–2.57	0.013
Knowing that problems in the global food supply were consequences of climate change	1.61	1.07–2.41	0.023
Being married/cohabitant	1.49	1.01–2.18	0.043
Good/very good self-assessed knowledge about climate change	1.27	0.83–1.94	0.257
Knowing that infants/children, the elderly, and people with multiple medical conditions are more sensitive to climate change	1.26	0.82–1.94	0.283
Having a postgraduate degree (master, PhD)	1.22	0.83–1.79	0.300
**Variable**	**OR**	**95% CI**	***p***
**+ Model 5. Believing information campaigns and Public Health emergency plans are very important to reduce climate change** Log likelihood = −353.63698, χ^2^ = 45.43 (10 df), *p* < 0.0001 (sample size 544)			
Elderly HCWs	1.02	1.01–1.04	0.008
Knowing that infants/children, the elderly, and people with multiple medical conditions are more sensitive to climate change	1.69	1.12–2.55	0.013
Females	1.58	1.08–2.29	0.017
Knowing that problems in the global food supply were consequences of climate change	1.48	1.01–2.18	0.048
*Ward type*			
Emergency/Resuscitation/Intensive care	0.66	0.42–1.01	0.057
Surgery/Operating Room	Backward elimination		
Clinical Departments	Reference category		
Nurses	1.45	0.96–2.19	0.076
Good/very good self-assessed knowledge about climate change	1.39	0.92–2.09	0.113
Receiving information about climate change from scientific journals	1.28	0.89–1.85	0.183
Knowing that the spread of infectious diseases was a consequence of climate change	1.24	0.83–1.85	0.294
Needing additional information on climate change	1.23	0.77–1.95	0.390
**Variable**	**OR**	**95% CI**	***p***
**@ Model 6. Educating patients for improving sustainability and health protection** Log likelihood = -340.43596, χ^2^ = 64.09 (11 df), *p* < 0.0001 (sample size 543)			
Having received information about climate change in training courses	2.81	1.71–4.61	<0.001
Males	0.61	0.41–0.89	0.012
Knowing that infants/children, the elderly, and people with multiple medical conditions are more sensitive to climate change	1.71	1.12–2.61	0.013
Being very scared of climate change	1.64	1.11–2.43	0.013
Having at least one chronic disease	1.77	1.08–2.91	0.024
Having a postgraduate degree (master, PhD)	0.67	0.46–0.97	0.033
Receiving information about climate change from scientific journals	1.43	0.98–2.08	0.065
Younger HCWs	0.98	0.9 6–1.01	0.082
Strongly agree that climate change should be included in the training of HCWs	1.39	0.89–2.17	0.143
Needing additional information on climate change	1.39	0.87–2.21	0.165
Being unmarried/widowed/divorced	0.77	0.52–1.16	0.214

° The following variables were deleted by the stepwise procedure: Knowing that water scarcity or clean water conservation were consequences of climate change. * The following variables were deleted by the stepwise procedure: Needing additional information on climate change; having at least a chronic disease. + The following variables were deleted by the stepwise procedure: Knowing that water scarcity or clean water conservation were consequences of climate change. @ The following variables were deleted by the stepwise procedure: Believing that the formulation and implementation of laws and regulations in relation to combating climate change is extremely important; professional role; knowing that water scarcity or clean water conservation were consequences of climate change; knowing that the spread of infectious diseases was a consequence of climate change; knowing that problems in the global food supply were consequences of climate change; self-assessed knowledge about climate change; believing that it is extremely important to improve scientific research on useful interventions in addressing climate change.

**Table 3:** Number and percentages of participants identifying the proposed items as causes and consequences of climate change.

Causes of Climate Change N (%)		Consequences of Climate Change N (%)	
Serious air pollution	482 (85.5%)	Rising temperatures	479 (84.9%)
Destruction of forest and farmland	385 (68.3%)	Melting glaciers and rising sea levels	462 (81.9%)
Greenhouse effect	331 (58.7%)	Extreme weather events	381 (67.5%)
Ecological environment deterioration	268 (47.5%)	Natural disasters	347 (61.5%)
Rapid development of the industry	222 (39.4%)	Problems in the global food supply	233 (41.3%)
Natural changes in the atmosphere	185 (32.8%)	Spread of infectious diseases	205 (36.3%)
Population explosion	146 (25.9%)	Economic crises and social inequalities	182 (32.2%)
Increase in heat engine vehicles	173 (30.7%)		
Rural urbanization process	122 (21.6%)	Water scarcity or clean water conservation	87 (15.4%)
Discrepancy between environmental policies between East and West	1 (0.2%)	Biodiversity loss	77 (13.6%)

**Table 4:** Frequency of eco-sustainable activities.

	Never	Rarely	Sometimes	Often	Always
	N (%)	N (%)	N (%)	N (%)	N (%)
**Use public transport**	107 (19%)	108 (19.2%)	145 (25.7%)	127 (22.5%)	77 (13.6%)
**Walking or cycling to work**	205 (36.4%)	59 (10.5%)	79 (14%)	117 (20.7%)	104 (18.4%)
**Turn off lights and/or devices when not in use**	13 (2.3%)	19 (3.4%)	70 (12.4%)	137 (24.3%)	325 (57.6%)
**Buying energy-saving light bulbs**	12 (2.1%)	12 (2.1%)	74 (13.1%)	116 (20.6%)	350 (62.1%)
**Turn off the taps if water is not needed**	5 (0.9%)	8 (1.4%)	48 (8.5%)	90 (16%)	413 (73.2%)
**Reduce the use of plastic/recycle it when possible**	2 (0.3%)	19 (3.4%)	71 (12.6%)	153 (27.1%)	319 (56.6%)
**Buying environmentally friendly products**	2 (0.3%)	24 (4.3%)	132 (23.4%)	173 (30.7%)	233 (41.3%)
**Using organic products for food**	17 (3%)	59 (10.5%)	168 (29.8%)	158 (28%)	162 (28.7%)
**Sharing your car with friends/colleagues while traveling**	79 (14%)	56 (9.9%)	136 (24.1%)	139 (24.7%)	154 (27.3%)

## Data Availability

The data presented in this study are available upon request from the corresponding author.

## Supplementary Materials

The following supporting information can be downloaded at: https://www.mdpi.com/article/10.3390/healthcare14111576/s1, Supplementary File S1: The independent variables included in the different final models. Supplementary File S2: HCWs attitudes on climate change.
