# Supplementary material for: Climate Change and Sustainable Healthcare: Knowledge, Attitudes, and Educational Role of Healthcare Workers
Source: Healthcare (Basel). 2026 Jun 4;14(11):1576. doi: 10.3390/healthcare14111576 (PMC13256475; doi:10.3390/healthcare14111576)
Supplement: Supplementary file 1 [file healthcare-14-01576-s001.zip › File S1.pdf]

Supplementary File S1

The independent variables included in the different final models.

Model	Outcome	Independent variables
**1**	Self-assessed knowledge about climate change (dichotomous) (poor/low/moderate = 0; good/very good = 1)	• Gender (male=0; female=1)<br>• Age in years (continuous)<br>• Receiving information about climate change from scientific journals (no=0; yes=1)<br>• Needing additional information on climate change (no=0; yes=1)
**2**	Considering climate change an urgent problem (dichotomous) (no = 0; yes = 1)	• Gender (male=0; female=1)<br>• Professional role (others=0; nurse=1)<br>• Ward type (emergency/resuscitation/intensive care=1; surgery/operating room=2; clinical departments=3)<br>• Chronicdisease (no=0; yes=1)<br>• Self-assessed knowledge about climate change (dichotomous) (poor/low/moderate=0; good/very good=1)<br>• Knowing that water scarcity or clean water conservation were consequences of climate change (no=0; yes=1)<br>• Knowing that the spread of infectious diseases was a consequence of climate change (no=0; yes=1)<br>• Knowing that problems in the global food supply were consequences of climate change (no=0; yes=1)<br>• Knowing that infants/children, the elderly, and people with multiple medical conditions are more sensitive to climate change (no=0; yes=1)<br>• Having received information about climate change from scientific journals (no=0; yes=1)<br>• Needing additional information on climate change (no=0; yes=1)
**3**	Believing that climate change will negatively affect global health (dichotomous) (strongly disagree/disagree/uncertain/agreed = 0; strongly agree = 1)	• Gender (male=0; female=1)<br>• Educational level (not postgraduate degree=0; postgraduate degree (master, PhD)=1)<br>• Chronic disease (no=0; yes=1)<br>• Self-assessed knowledge about climate change (dichotomous) (poor/low/moderate=0; good/very good=1)

		• Knowing that water scarcity or clean water conservation were consequences of climate change (no=0; yes=1)<br>• Knowing that the spread of infectious diseases was a consequence of climate change (no=0; yes=1)<br>• Knowing that problems in the global food supply were consequences of climate change (no=0; yes=1)<br>• Knowing that infants/children, elderly, and people with multiple medical conditions are more sensitive to climate change (no=0; yes=1)<br>• Needing additional information on climate change (no=0; yes=1)
**4**	Believing that climate change is causing health problems (dichotomous) (no = 0; yes = 1)	• Gender (male=0; female=1)<br>• Educational level (not postgraduate degree = 0; postgraduate degree (master, PhD)=1)<br>• Marital status (unmarried/widowed/divorced=0; married/cohabitant=1)<br>• Chronic disease (no=0; yes=1)<br>• Self-assessed knowledge about climate change (dichotomous) (poor/low/moderate=0; good/very good=1)<br>• Knowing that water scarcity or clean water conservation were consequences of climate change (no=0; yes=1)<br>• Knowing that the spread of infectious diseases was a consequence of climate change (no=0; yes=1)<br>• Knowing that problems in the global food supply were consequences of climate change (no=0; yes=1)<br>• Knowing that infants/children, the elderly, and people with multiple medical conditions are more sensitive to climate change (no=0; yes=1)<br>• Needing additional information on climate change (no=0; yes=1)
**5**	Believing information campaigns and Public Health emergency plans are very important to reduce climate change (dichotomous) (no = 0; yes = 1)	• Gender (male=0; female=1)<br>• Age in years (continuous)<br>• Professional role (others=0; nurse=1)<br>• Ward type (emergency/resuscitation/intensive care=1; surgery/operating room=2; clinical departments=3)

		• Self-assessed knowledge about climate change (dichotomous) (poor/low/moderate=0; good/very good=1)<br>• Knowing that water scarcity or clean water conservation were consequences of climate change (no=0; yes=1)<br>• Knowing that the spread of infectious diseases was a consequence of climate change (no=0; yes=1)<br>• Knowing that problems in the global food supply were consequences of climate change (no=0; yes=1)<br>• Knowing that infants/children, the elderly, and people with multiple medical conditions are more sensitive to climate change (no=0; yes=1)<br>• Having received information about climate change from scientific journals (no=0; yes=1)<br>• Needing additional information on climate change (no=0; yes=1)
**6**	Educating patients for improving sustainability and health protection (dichotomous) (no = 0; yes = 1)	• Gender (male=0; female=1)<br>• Age in years (continuous)<br>• Professional role (others=0; nurse=1)<br>• Educational level (not postgraduate degree = 0; postgraduate degree (master, PhD)=1)<br>• Marital status (unmarried/widowed/divorced=0; married/cohabitant=1)<br>• Chronic disease (no=0; yes=1)<br>• Self-assessed knowledge about climate change (dichotomous) (poor/low/moderate=0; good/very good=1)<br>• Knowing that water scarcity or clean water conservation were consequences of climate change (no=0; yes=1)<br>• Knowing that the spread of infectious diseases was a consequence of climate change (no=0; yes=1)<br>• Knowing that problems in the global food supply were consequences of climate change (no=0; yes=1)<br>• Knowing that infants/children, the elderly, and people with multiple medical conditions are more sensitive to climate change (no=0; yes=1)<br>• Being very scared of climate change (no=0; yes=1)<br>• Strongly agree that climate change should be included in the training of HCWs (no=0; yes=1)

		• Believing extremely important the formulation and implementation of laws and regulations in relation to combating climate change (no=0; yes=1)<br>• Believing extremely important to improve scientific research on useful interventions in addressing climate change (no=0; yes=1)<br>• Having received information about climate change from scientific journals (no=0; yes=1)<br>• Having received information about climate change in training courses (no=0; yes=1)<br>• Needing additional information on climate change (no=0; yes=1)
