# Supplementary material for: Climate Change and Sustainable Healthcare: Knowledge, Attitudes, and Educational Role of Healthcare Workers
Source: Healthcare (Basel). 2026 Jun 4;14(11):1576. doi: 10.3390/healthcare14111576 (PMC13256475; doi:10.3390/healthcare14111576)
Supplement: Supplementary file 1 [file healthcare-14-01576-s001.zip › File S2.pdf]

Supplementary File S2. HCW's attitudes on climate change.

Level of agreement/disagreement on climate change					
	**Strongly disagree**	**Disagree**	**Not sure**	**Agree**	**Strongly agree**
	**N(%)**	**N(%)**	**N(%)**	**N(%)**	**N(%)**
Climate change is happening	12(2.1%)	14(2.5%)	56(9.9%)	155(27.5%)	327(58%)
Climate change is an inevitable consequence of our society	37(6.6%)	61(10.8%)	151(26.8%)	166(29.4%)	149(26.4%)
Climate change is a natural variation of the temperature	134(23.8%)	151(26.8%)	128(22.6%)	88(15.6%)	63(11.2%)
Climate change will have negative effects on the Italian population	8(1.4%)	15(2.7%)	76(13.5%)	189(33.5%)	276(48.9%)
The consequences of climate change scare me	17(3%)	19(3.4%)	97(17.2%)	162(28.7%)	269(47.7%)
Human activities are the main cause of climate change	10(1.8%)	20(3.6%)	108(19.1%)	202(35.8%)	224(39.7%)
It is too late to take action on climate change	95(16.8%)	127(22.5%)	225(39.9%)	78(13.8%)	39(6.9%)
**Climate change consequences on the future health needs of the population**					
	**Strongly disagree**	**Disagree**	**Not sure**	**Agree**	**Strongly agree**
	**N(%)**	**N(%)**	**N(%)**	**N(%)**	**N(%)**
It will cause global health problems	5(0.9%)	12(2.1%)	102(18.1%)	212(37.6%)	233(41.3%)
It will be a problem of care relevance for HCWs	14(2.5%)	37(6.6%)	180(31.9%)	221(39.2%)	112(19.8%)
The topic should be included in the training of HCWs	14(2.5%)	44(7.8%)	174(30.8%)	178(31.6%)	154(27.3%)
HCWs' actions can reduce the impact of climate change	51(9%)	101(17.9%)	209(37.1%)	140(24.8%)	63(11.2%)
HCWs have the capabilities to deal with the consequences of climate change	79(14%)	169(30%)	206(36.5%)	76(13.5%)	34(6%)
HCWs are ready to address the consequences of climate change	113(20%)	174(30.9%)	196(34.7%)	54(9.6%)	27(4.8%)
**Climate change as a cause of future health-related problems**					
	**Strongly disagree**	**Disagree**	**Not sure**	**Agree**	**Strongly agree**
	**N(%)**	**N(%)**	**N(%)**	**N(%)**	**N(%)**
Infectious diseases caused by vectors (e.g. insects, mosquitoes, etc.)	11(2%)	32(5.7%)	135(23.9%)	215(38.1%)	171(30.3%)
Diarrhea caused by foodborne illnesses or carried by water following floods or inundations (Salmonella, Giardia, etc.)	8(1.4%)	36(6.4%)	117(20.7%)	231(41%)	172(30.5%)
Consequences on mental health status (anxiety, depression)	6(1.1%)	28(5%)	130(23%)	211(37.4%)	189(33.5%)
Food/water deficiency diseases (e.g. malnutrition, dehydration	11(2%)	17(3%)	124(22%)	215(38.1%)	194(34.9%)
Disruption of health services during extreme weather phenomena	40(7.1%)	49(8.7%)	145(25.7%)	189(33.5%)	141(25%)
Diseases related to extreme heat or cold (cardiovascular or dermatological diseases, heat stroke, cardiovascular diseases)	10(1.8%)	25(4.4%)	137(24.3%)	218(38.7%)	174(30.8%)
Air pollution increasing the severity of diseases (asthma, COPD, cardiovascular disease)	5(0.9%)	7(1.2%)	84(14.9%)	213(37.8%)	255(45.2%)
Serious injuries following landslides, floods and fires	3(0.5%)	18(3.2%)	116(20.6%)	216(38.3%)	211(37.4%)

Increased treatment for allergic sensitization and symptoms from exposure to plants and moulds (allergic symptoms)	6(1.1%)	25(4.4%)	143(25.3%)	199(35.3%)	191(33.9%)
**Actions to reduce climate change**					
	**Not at all important**	**Slightly important**	**Moderately important**	**Very important**	**Extremely important**
	**N(%)**	**N(%)**	**N(%)**	**N(%)**	**N(%)**
Control of greenhouse gas emissions	9(1.6%)	8(1.4%)	77(13.7%)	145(25.7%)	325(57.6%)
Creation of Public Health emergency plans	8(1.4%)	12(2.2%)	83(14.7%)	162(28.7%)	299(53%)
Formulation and implementation of laws and regulations in relation to combating climate change	11(1.9%)	5(0.9%)	64(11.6%)	171(30.3%)	313(55.5%)
Improving scientific research on useful interventions in addressing climate change	7(1.3%)	8(1.4%)	73(12.9%)	165(29.3%)	311(55.1%)
Information campaigns	8(1.4%)	7(1.3%)	59(10.5%)	139(24.6%)	351(62.2%)
